# Entropy exchange for infinite-dimensional systems

**DOI:** 10.1038/srep41692

**Published:** 2017-02-06

**Authors:** Zhoubo Duan, Jinchuan Hou

**Affiliations:** 1 Department of Mathematics, Taiyuan University of Technology, Taiyuan 030024, P. R. of China

## Abstract

In this paper the entropy exchange for channels and states in infinite-dimensional systems are defined and studied. It is shown that, this entropy exchange depends only on the given channel and the state. An explicit expression of the entropy exchange in terms of the state and the channel is proposed. The generalized Klein’s inequality, the subadditivity and the triangle inequality about the entropy including infinite entropy for the infinite-dimensional systems are established, and then, applied to compare the entropy exchange with the entropy change.

In quantum mechanics a quantum system is associated with a separable complex Hilbert space *H*. A quantum state *ρ* is a density operator, that is, 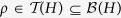 which is positive and has trace 1, where 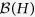 and 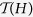 denote the von Neumann algebras of all bounded linear operators and the space of all trace-class operators with 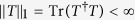, respectively. Let us denote by 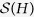 the set of all states in the quantum system associated with *H*. A state *ρ* is called a pure state if *ρ*^2^ = *ρ*; otherwise, *ρ* is called a mixed state.

Consider two quantum systems associated with Hilbert spaces *H* and *K* respectively. Recall that a quantum channel between these two systems is a trace-preserving completely positive linear map from 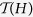 into 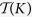. It is known^1^,^2^,^3^,^4^ that every channel 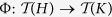 has an operator-sum representation


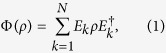


where 1 ≤ *N* ≤ ∞ and 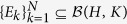 is a sequence of bounded linear operators from *H* into *K* with 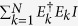. *E*_*k*_*s* are called the operation elements or Kraus operators of the quantum channel Φ. The representation of Φ in Eq. (1) is not unique. If both *H* and *K* are finite-dimensional, it is well known that *N* ≤ dim *H* dim *K* < ∞ and the sequences 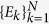 and 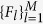 of operation elements of any two representations of Φ are connected by a unitary matrix, 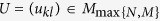 such that 
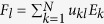
, 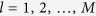. This fact is so-called the unitary freedom in the operator-sum representation for quantum channels. However, unitary freedom is no longer valid for infinite-dimensional systems^5^. In fact, what we have is so-called the bi-contractive freedom, which asserts that, if a channel 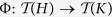 has two operator-sum representations 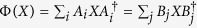 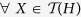, then there exist contractive matrices Ω = (*ω*_*ij*_) and Γ = (*γ*_*ji*_) such that 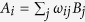 for each *i* and 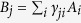 for each *j*. The converse is also true. Particularly, if Ω = (*ω*_*ij*_) is an isometry so that 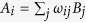 for each *i*, then 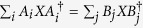 holds for any *X*.

Let *R* and *Q* be two quantum systems described by Hilbert spaces *H*_*R*_ and *H*_*Q*_, respectively. Suppose that the joint system *RQ* is prepared in a pure entangled state 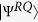 and the initial state of system *Q* is 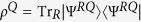. The system *R* is dynamically isolated and has a zero internal Hamiltonian, while the system *Q* undergoes some evolution that possibly involves interaction with the environment *E*. The final state of *RQ* is possibly mixed and is described by the density operator *ρ*^*RQ*′^. Thus, if the dynamical evolution that *Q* is subjected to is described by Φ^*Q*^, then the final state is 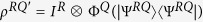 and the entanglement fidelity is refs 6, 7, 8, 9


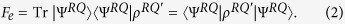


The value of *F*_*e*_ is independent of the choice 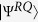 of purification of *ρ*^*Q*^. In fact, it was shown^5^,^7^,^10^,^11^ that for any 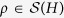 with dim *H* ≤ ∞ and any quantum channel Φ with operation elements {*E*_*i*_}, we have 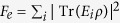.

For finite-dimensional systems there is another quantity concerning channels and states that is intrinsic to subsystem *Q*. This quantity is called the entropy exchange. For a given state *ρ*^*Q*^ and a given channel Φ^*Q*^ in a finite-dimensional system *Q*, recall that the entropy exchange *S*_*e*_ is defined by refs 1, 6 and 12, 13, 14


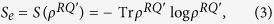


where 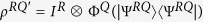 and 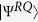 is a purification of *ρ*^*Q*^. It was shown^1^,^6^ that the entropy exchange *S*_*e*_ is independent of the choice of purification 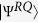 of the state *ρ*^*Q*^. It was also shown^1^ that the entropy exchange *S*_*e*_ has another explicit formulation


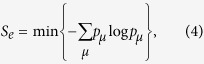


where 
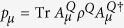
 with 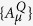 the sequence of the Kraus operators of an operator-sum representation of Φ^*Q*^ and the minimum is taken over all operator-sum representations of Φ^*Q*^.

It is clear that Eq. (3) can be naturally generalized to infinite-dimensional case to give a definition of the entropy exchange for channels and states in infinite-dimensional systems. In continuous variable systems, Chen and Qiu^15^ studied the coherent information *I*_*e*_ = *S*(*ρ*^*Q*^) − *S*_*e*_ of the thermal radiation signal *ρ*^*Q*^ transmitted over the thermal radiation noise channel, one of the most essential quantum Gaussian channels, and derived an analytical expression for computation of the value of it. However, as the von Neumann entropy *S*(*ρ*) of a non-Gaussian state in an infinite-dimensional system may be +∞^16^, we may have *S*_*e*_ = +∞. In this paper we consider general states and channels and show that the definition Eq. (3) does not depend on the choice of the purification of the state either, and Eq. (4) is still true for infinite-dimensional systems.

For finite-dimensional systems, it is known^6^ that the entropy exchange is larger than or equal to the change of the entropy, that is,


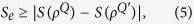


where *ρ*^*Q*′^ = Φ^*Q*^(*ρ*^*Q*^). The second purpose of the present paper is to compare the entropy exchange with the change of the entropy and to check whether or not the inequality (5) is still valid in infinite-dimensional systems. We show that, for infinite-dimensional case, what we can have are the following three inequalities: 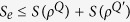, 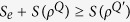 and 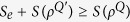. Thus, if both *S*(*ρ*^*Q*^) and *S*(*ρ*^*Q*′^) are finite, we still have 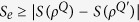. To prove the above inequalities, we need the subadditivity and the triangle inequality of von Neumann entropies for infinite-dimensional quantum systems. These two inequalities were established in a more general frame of von Neumann algebras for normal states with finite entropy^17^. However, for the convenience of readers, we present some elementary proofs including the case of infinite von Neumann entropy here by establishing the generalized Klein’s inequality for infinite-dimensional case. We also give some examples which illustrates that the entropy exchange is different from the change of entropy.

## Entropy exchange for infinite-dimensional systems

In this section, we mainly give some properties of the entropy exchange for infinite-dimensional systems. In fact, the results in this section hold for both finite- and infinite-dimensional cases.

Recall that a linear operator *U* from a Hilbert space into another is called an isometry if 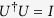; a coisometry if 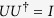. Obviously, if the spaces are finite-dimensional with the same dimension, isometries and coisometries are unitary operators.

**Lemma 1.** *Suppose* |*ϕ*〉 *and* |*ψ*〉 *are two pure states of an infinite*-*dimensional composite system with subsystems R and Q*. *If they have identical Schmidt coefficients*, *then there are isometries or coisometries U on system R and V on system Q such that* 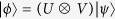.

**Proof.** By the assumption, |*ϕ*〉 and |*ψ*〉 have respectively the Schmidt decompositions 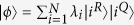 and 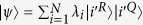, where 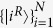 and 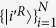 are two orthonormal sets for system *R*, 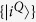 and 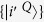 are two orthonormal sets for system *Q*, *λ*_*i*_ > 0 with 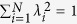. Extend 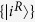 to an orthonormal basis 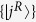

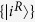
, 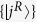 and 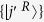 to an orthonormal basis {|*i*′^*R*^〉, |*j*′^*R*^〉} of the system *R*. In the same way, extend {|*i*^*Q*^〉} to an orthonormal basis {|*i*^*Q*^〉, |*l*^*Q*^〉}, and {|*i*′^*Q*^〉} to an orthonormal basis {|*i*′^*Q*^〉, |*l*′^*Q*^〉} of the system *Q*. Denote the cardinal number of a set 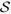 by 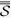. Let 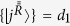, 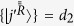, 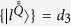 and 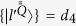. Clearly, we have 9 possible cases.

**Case 1.** *d*_1_ = *d*_2_ and *d*_3_ = *d*_4_. Let unitary operators *U* on system *R* and *V* on system *Q* be defined respectively by *U*|*i*^*R*^〉 = |*i*′^*R*^〉 for 1 ≤ *i* ≤ *N* and *U*|*j*^*R*^〉 = |*j*′^*R*^〉 for 1 ≤ *j* ≤ *d*_1_ = *d*_2_; *V*|*i*^*Q*^〉 = |*i*′^*Q*^〉 for 1 ≤ *i* ≤ *N* and *V*|*l*^*Q*^〉 = |*l*′^*Q*^〉 for 1 ≤ *l* ≤ *d*_*3*_ = *d*_4_. Then 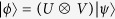.

**Case 2.** *d*_1_ = *d*_2_ and *d*_3_ < *d*_4_. Let *U* be defined as in Case 1 and *V* be defined by *V*|*i*^*Q*^〉 = |*i*′^*Q*^〉 for 1 ≤ *i* ≤ *N* and *V*|*l*^*Q*^〉 = |*l*′^*Q*^〉 for 1 ≤ *l* ≤ *d*_3_ < *d*_4_. Then *U* is a unitary operator on system *R* and *V* is an isometry *V* on system *Q* satisfying 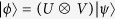.

**Case 3.** *d*_1_ = *d*_2_ and *d*_3_ > *d*_4_. Define *U* on system *R* as in Case 1 and define *V* on system *Q* by *V*|*i*^*Q*^〉 = |*i*′^*Q*^〉 for 1 ≤ *i* ≤ *N*, and *V*|*l*^*Q*^〉 = |*l*′^*Q*^〉 for 1 ≤ *l* ≤ *d*_4_ and *V*|*l*^*Q*^〉 = 0 for *d*_4_ < *l* ≤ *d*_3_. Then *U* is unitary and *V* is coisometric so that 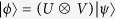.

In a similar way, it is obvious to see that

**Case 4.** *d*_1_ < *d*_2_ and *d*_3_ = *d*_4_. There is an isometry *U* on system *R* and a unitary *V* on system *Q* such that 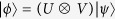.

**Case 5.** *d*_1_ < *d*_2_ and *d*_3_ < *d*_4_. There are isometries *U* on system *R* and *V* on system *Q* such that 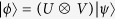.

**Case 6.** *d*_1_ < *d*_2_ and *d*_3_ > *d*_4_. There is an isometry *U* on system *R* and a coisometry *V* on system *Q* such that 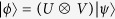.

**Case 7.** *d*_1_ > *d*_2_ and *d*_3_ = *d*_4_. There is a coisometry *U* on system *R* and a unitary *V* on system *Q* such that 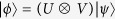.

**Case 8.** *d*_1_ > *d*_2_ and *d*_3_ < *d*_4_. There is a coisometry *U* on system *R* and an isometry *V* on system *Q* such that 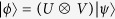.

**Case 9.** *d*_1_ > *d*_2_ and *d*_3_ > *d*_4_, there are coisometries *U* on system *R* and *V* on system *Q* such that 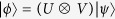. ◽

**Lemma 2.** *If* 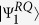 *and*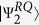 *are purifications of a state ρ*^*Q*^ *to a composite system RQ*, *then there exists an isometry V*^*R*^ *on system R such that either* 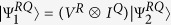 *or* 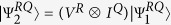.

**Proof.** Let 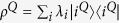 be the spectral decomposition of *ρ*^*Q*^ with *λ*_*i*_ ≥ *λ*_*i*+1_. Since both 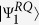 and 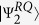 are purifications of *ρ*^*Q*^, their Schmidt decompositions have the form 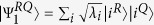 and 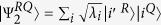, where 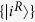 and 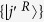 are two orthonormal sets for system *R*. Hence 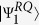 and 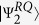 have identical Schmidt coefficients. Making use of lemma 1, there is an isometry or a coisometry *U*^*R*^ on system *R* such that 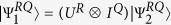. If *U*^*R*^ is already an isometry, we have done. If *U*^*R*^ is a coisometry, by the proof of Lemma 1 we see that there is an isometry *V*^*R*^ such that 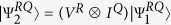 and 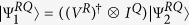.◽

**Lemma 3.** *Assume that* 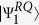 *and*  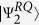 *are two purifications of a state ρ*^*Q*^ *to a composite system RQ*, *and each is subjected to the same evolution superoperator* 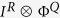 *with the resulting states respectively* 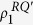 *and* 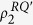, *i*.*e*., 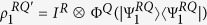 *and* 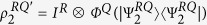. *Then there exists an isometry V*^*R*^ *on system R such that either* 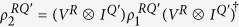 *or* 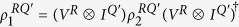.

**Proof.** By lemma 2, there exists an isometry transformation *V*^*R*^ acting on system *R* such that either 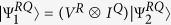 or 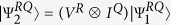. Without loss of generality, assume that 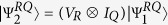. Let 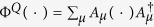 be an operator-sum representation of Φ^*Q*^. Then


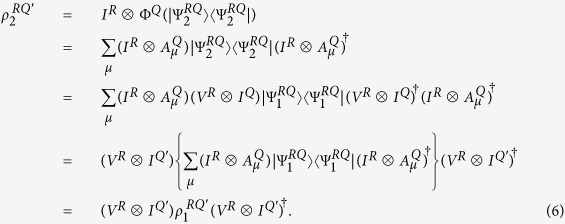


Similarly, if 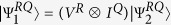 holds, then we have


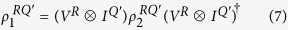


◽

**Lemma 4.** *If A is a bounded self*-*adjoint operator on a complex Hilbert space and f is a continuous function on σ*(*A*), *the spectrum of A*, *then*, *for any isometric operator V*, *we have* 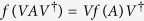.

**Proof.** As *A* is a bounded self-adjoint operator, 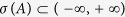 is a bounded closed set. Because *f* is a continuous function on *σ*(*A*), we can apply the Weierstrass theorem to find a sequence of polynomials {*P*_*n*_} such that *P*_*n*_ → *f* uniformly on *σ*(*A*). Write 
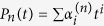
. It is clear that 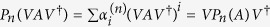 since *V* is an isometric operator. Let *n*→∞, we see that 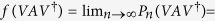

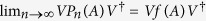
.

The following result reveals that, for infinite-dimensional systems, similar to the entanglement fidelity^5^, the value of entropy exchange is also independent of the choice of purifications of the initial state.

**Theorem 5.** *The entropy exchange of a channel* Φ^*Q*^ *and a state ρ*^*Q*^ *is independent of the choice of purifications of the state ρ*^*Q*^.

**Proof.** Let 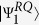 and 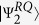 be two purifications of the state *ρ*^*Q*^ in composite system *RQ*, and denote 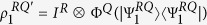 and 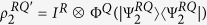. By the definition Eq. (3), we have to show that


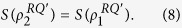


By lemma 3, there is an isometry *V*^*R*^ so that the resulting states 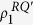 and 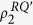 satisfy either 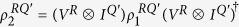 or 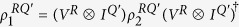. Without loss of generality, suppose 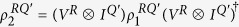. Note that *f*(*x*) = *x* log *x* is a continuous function on 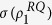. Then, by lemma 4,


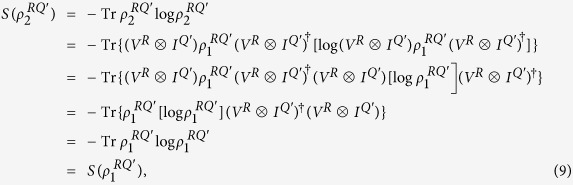


as desired.◽

In the sequel, analogue to Eq. (4) for finite-dimensional systems, we derive an explicit expression for *S*_*e*_ in terms of *ρ*^*Q*^ and Φ^*Q*^ for infinite-dimensional systems.

To do this, we need some more lemmas.

**Lemma 6.** *Let* 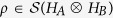 *with* 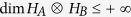. *For any* 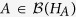 *and* 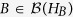, *we have* 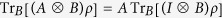 *and* 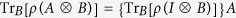.

**Proof.** Fix an orthonormal basis {|*i*〉} of *H*_*B*_. Then *B* can be written in a matrix *B* = (*b*_*ij*_), and 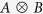 and *ρ* can be written in operator matrices 
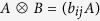
 and *ρ* = (*ρ*_*ij*_), respectively. Thus we have 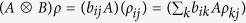, and then


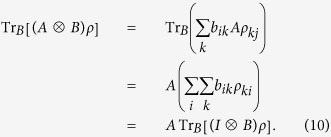


Similarly, we can drive that


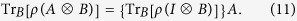


**Lemma 7.** *Let* 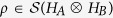 *with* 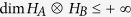. *Then*, *for any* 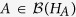 *and* 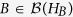, *we have*


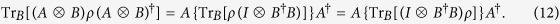


**Proof.** By lemma 6 and with the same symbols as in the proof of lemma 6, we have


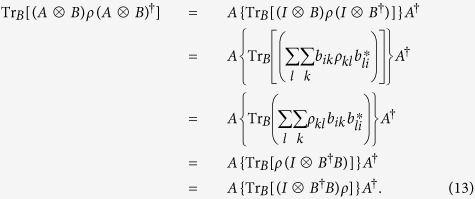


◽

Let Φ^*Q*^ be a channel from system *Q* into system *Q*′. Suppose 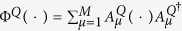 (*M* ≤ ∞) is an operator-sum representation for the channel Φ^*Q*^. If *ρ*^*Q*^ is a state of system *Q* and 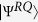 is a purification of *ρ*^*Q*^ into composite system *RQ*, then, for any *μ*, let 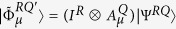. Thus the resulting state *ρ*^*RQ*′^ can be written in


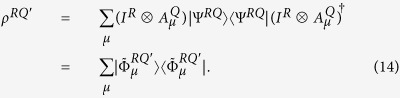


Therefore 
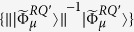
 is a pure state ensemble for *ρ*^*RQ*′^. Let us adjoin a system *E* with Hilbert space *H*_*E*_, where dim *H*_*E*_ = *M*. Then, for any orthonormal basis 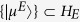, the state 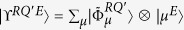 is a purification of *ρ*^*RQ*′^. With these symbols, we have

**Lemma 8.** *Let* 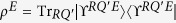. *Then we have S*_*e*_ = *S*(*ρ*^*E*^).

**Proof.** Since the state 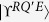 is a pure state, the reduced states 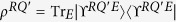 and 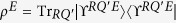 have the same von Neumann entropy. Therefore, by the definition of the exchange entropy, we get 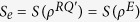.◽

Furthermore, let us write down the density operator *ρ*^*E*^ in matrix form. Clearly,


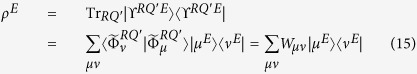


with 
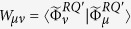
. By lemmas 6 and 7, we see that


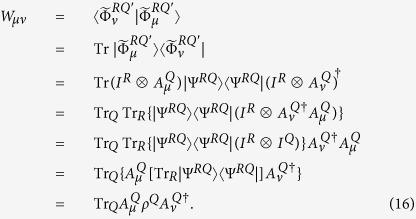


Let *W* be the density operator with components 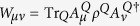. Then, by lemma 8, *S*_*e*_ = *S*(*W*). Now, let 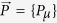 with *P*_*μ*_ = *W*_*μμ*_. Thus 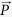 is a probabilities which is given by the state *W* from a complete measurement using the basis that yields the matrix elements *W*_*μν*_. Therefore we have 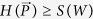 as measurements increasing the entropy.

Now, we are at a position to give an explicit formula for the entropy exchange based upon the operator-sum representation for quantum channel Φ^*Q*^ and the initial state *ρ*^*Q*^ for an infinite-dimensional system.

**Theorem 9.** Let 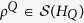 be a state with dim *H*_*Q*_ ≤ ∞ and 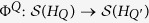 a channel. Then the entropy exchange


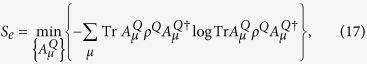


◽where 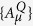 is a sequence of Kraus operators of an operator-sum representation of Φ^*Q*^, that is, 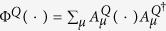, and the minimum is taken over all operator-sum representations of Φ^*Q*^.

**Proof.** For given state *ρ*^*Q*^ and quantum channel Φ^*Q*^, if {*A*_*μ*_} is the sequence of Kraus operators of an operator-sum representation of Φ^*Q*^, then by lemma 8 and the discussion previous theorem 9, 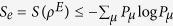, where, 
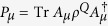
, 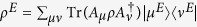 for some orthonormal basis {|*μ*^*E*^〉} for the environment system *E*. Hence we have 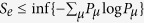. In the sequel we show that 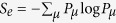 for some suitable choice of operator-sum representation of Φ^*Q*^. In fact, for a given sequence {*A*_*μ*_} of Kraus operators for an operator-sum representation of Φ^*Q*^, 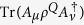
s are the matrix elements of *ρ*^*E*^ in the orthonormal basis {|*μ*^*E*^〉}. Let *W* be the associated matrix with entries 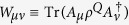, that is, *W* is the matrix of *ρ*^*E*^ in an appropriate basis; then *S*_*e*_ = *S*(*W*). Since *W* is a matrix representation of the environmental density operator, it may be diagonalized by a unitary matrix *U* = (*u*_*μν*_), i.e., 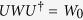, where 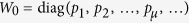 is a diagonal matrix. Letting |*μ*′^*E*^〉 = *U*|*μ*^*E*^〉, we have *ρ*^*E*^ = *W*_0_ in the basis {|*μ*′^*E*^〉}. Thus 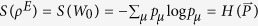. Now let 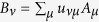; then, due to the theorem 2.1 in the paper^5^, {*B*_*ν*_} is a sequence of Kraus operators for an operator-sum representation of the quantum channel Φ^*Q*^, i.e. 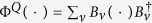. Moreover, 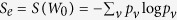 with obviously 
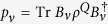
. So we have


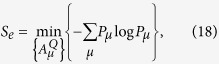


where 
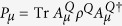
 and the minimum is taken over all operator-sum representations of Φ^*Q*^.◽

## Comparison with entropy change

The entropy exchange *S*_*e*_ simply characterizes the information exchange between the system *Q* and the external world during the evolution given by Φ^*Q*^. It is interesting to explore the relationship between the entropy exchange and the entropy change during the same evolution. Such a question was studied for finite-dimensional systems and the inequality (5) was established^6^. However, the inequality (5) does not always valid in infinite-dimensional case. To solve the question for infinite-dimensional systems, we need the subadditivity and the triangle inequality of von Neumann entropies for infinite-dimensional systems which was established in the textbook^17^ for normal states *with finite entropy* in a more general frame of von Neumann algebras. However, we have to deal with the states with infinite entropy. Here we present somewhat elementary proofs for these two inequalities by generalizing the generalized Klein’s inequality from finite-dimensional systems to the infinite-dimensional systems and clarify when the inequalities are still valid for states with infinite entropy.

Let 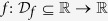 be a function. The following lemma 10 and 11 are obvious^18^.

**Lemma 10.** *If f is a convex* (*concave*) *function*, *then f is continuous*.

**Lemma 11.** *If f is a convex* (*concave*) *function*, *then f*(*y*) − *f*(*x*) ≥ (≤)(*y* − *x*) *f*′(*x*).

**Lemma 12.** *Suppose f is a convex* (*concave*) *function and A is a bounded self*-*adjoint operator on a Hilbert space H with* 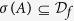. *If* 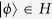 *is an unit vector*, *then* 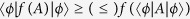.

**Proof.** By lemma 10, *f* is continuous. Let 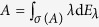 be the spectral decomposition of the self-adjoint operator *A*. Assume that *f* is convex. For any unit vector 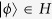, denote by *μ* the probability measure defined by 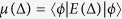 for any Borel set Δ. With {Δ_*k*_} any finite Borel partition of *σ*(*A*) and 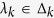, we have


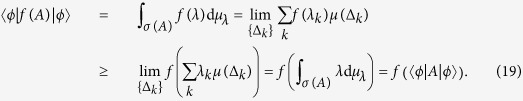


Similarly, if *f* is concave, then one gets


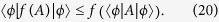


◽

**Lemma 13.** *Suppose f is a convex* (*concave*) *function*. *If A*, *B are two positive operators acting on a Hilbert space H and A is of trace*-*class*, *then*


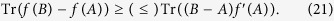


**Proof.** As *A* is a positive operator of trace-class, by spectral theorem, there exists an orthnormal basis 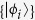 of *H* and nonnegative numbers *λ*_*i*_ such that 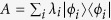. If *f* is convex, then by lemma 12 and lemma 11 we have


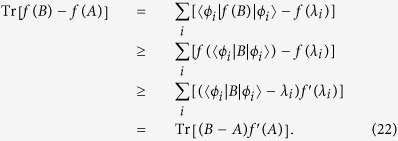


Similarly, if *f* is concave, then


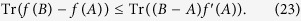


◽

In finite-dimensional case, the following result is valid and is called the generalized Klein’s inequality. We generalize it to infinite-dimensional case.

**Lemma 14.** (Generalized Klein’s inequality) *Let A*, *B be two positive operators of trace*-*class on a Hilbert space H*. *If* 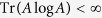, *then*


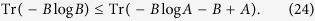


**Proof.** Take *f* so that *f*(*x*) = −*x* log *x* for *x* > 0 and *f*(0) = 0. Then *f*(*x*) is a concave function with 
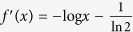
 and 
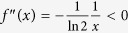
 for *x* > 0. By lemma 13, we have◽


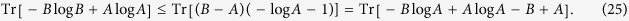


Since Tr*A* log *A* < ∞, we get 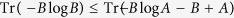, as desired.

Making use of this result, we see that the relative entropy 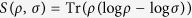 is also non-negative for the infinite-dimensional quantum systems whenever *S*(*σ*) < ∞.

**Corollary 15.** For any two density operators *ρ*, 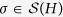, if Tr(*σ* log *σ*) < ∞, then


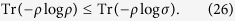


**Proof.** Since *ρ*, *σ* are two density operators, Tr *ρ* = Tr *σ* = 1. Substituting these in the inequality (24), we have 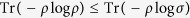.◽

Next, we apply the corollary 15 to prove the subadditivity inequality (27) and the triangle inequalities (29) and (30) for Von Neumann entropy.

**Lemma 16.** *Let* 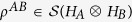 *be a state with* 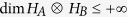. *Then*


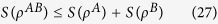


where *ρ*^*A*^ = Tr_*B*_*ρ*^*AB*^ and *ρ*^*B*^ = Tr_*A*_*ρ*^*AB*^.

**Proof.** Let *ρ* = *ρ*^*AB*^ and 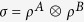. Then, 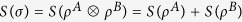. Note that


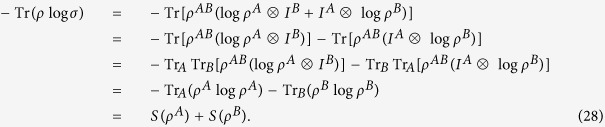


If *S*(*σ*) < ∞, corollary 15 and the above equations imply 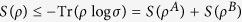. If *S*(*σ*) = ∞, then *S*(*ρ*^*A*^) + *S*(*ρ*^*B*^) = ∞, and obviously *S*(*ρ*^*AB*^) ≤ *S*(*ρ*^*A*^) + *S*(*ρ*^*B*^) holds.◽

In finite-dimensional case, the inequalities 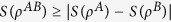 holds for any bipartite states and is called the triangle inequality. In infinite-dimensional case, this inequality may be not valid except the case when both *S*(*ρ*^*A*^), *S*(*ρ*^*B*^) are finite. What we can have is the triangle inequalities of the following kind.

**Lemma 17.** *Let* 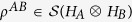 *with* 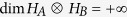. *Then*


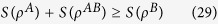


*and*


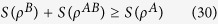


*where* 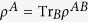, *and* 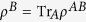.

**Proof.** To prove the inequality (29), we introduce a system *C* which purifies the system *AB*. Let 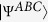 be a purification of *ρ*^*AB*^; then


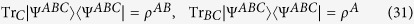


and


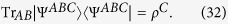


Applying the subadditivity, that is, lemma 16, we have


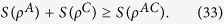


Since 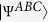 is a pure state, *S*(*ρ*^*AB*^) = *S*(*ρ*^*C*^) and *S*(*ρ*^*AC*^) = *S*(*ρ*^*B*^). Hence the previous inequality is the same as 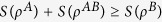.

By symmetry between the systems *A* and *B* one sees that 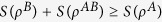 is also true.◽

Now, we relate the entropy exchange to change in the entropy of the system *Q* for infinite-dimensional quantum systems.

**Theorem 18.** *For any evolution* Φ^*Q*^ *and initial state ρ*^*Q*^ *in an infinite*-*dimensional system Q*, *with ρ*^*Q*′^ = Φ^*Q*^(*ρ*^*Q*^), *the following inequalities are true*.


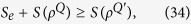



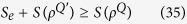


*and*


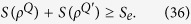


**Proof.** The evolution Φ^*Q*^ in fact is due to a unitary evolution of a larger system that includes an environment *E* with a pure initial state |0^*E*^〉 and the joint initial state 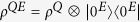. Obviously, we have *S*(*ρ*^*QE*^) = *S*(*ρ*^*Q*^). Since the joint system *QE* evolves unitarily, say 
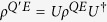
, one sees that 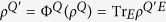 and the entropy of the joint state remains unchanged. Thus we have 
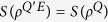
. Let 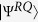 be a purification of *ρ*^*Q*^ to a larger system *RQ*; then 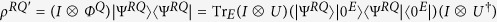. This means that 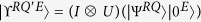 is a purification of *ρ*^*RQ*′^. Let 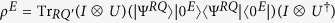. Then by the lemma 8, the entropy exchange *S*_*e*_ = *S*(*ρ*^*E*^). Using the inequality (27), one gets 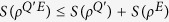, which gives 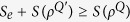. Applying the inequality (30), we obtain 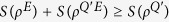, which entails 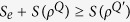. The inequality (29) implies that 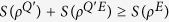, which establishes 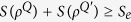.◽

By theorem 18 we known that 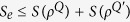 is always true. And, if both *S*(*ρ*^*Q*^), *S*(*ρ*^*Q*′^) are finite, then, as in finite-dimensional case, we have 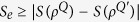, which means that the entropy exchange is not less than the change in entropy of the system *Q*. In general, the entropy exchange is different from the change in entropy of the system *Q*, that is, 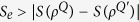 holds for some channels and states.

### Examples

The following is an example for finite-dimensional case.

**Example 1.** Let 
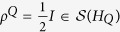
 with dim *H*_*Q*_ = 2. The bit flip channel Φ^*Q*^ flips the state of a qubit from |0〉 to |1〉 with probability 1 − *p*. It has operation elements


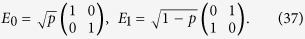


After some calculation, 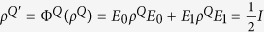, thus 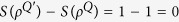.

On the other hand, note that 
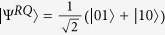
 is a purifications of *ρ*^*Q*^ to a composite system *RQ*, where dim *H*_*R*_ = 2. Thus


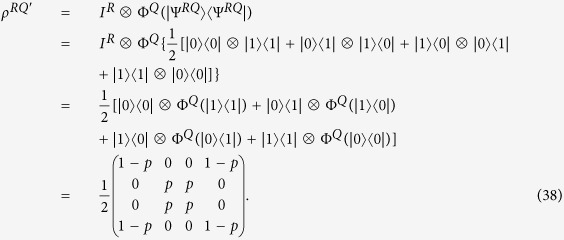


Obviously, the nonzero eigenvalues of *ρ*^*RQ*′^ are *p* and 1 − *p*, and thus, 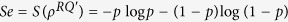. Hence we have 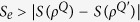 whenever 0 < *p* < 1.

Next we give an example for infinite-dimensional case.

**Example 2.** Consider the thermal radiation signal *ρ*^*Q*^ on a Gaussian system *Q*, which has Glauber’s P representation 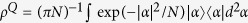. Here *N* is the average number of photons of *ρ*^*Q*^, |*α*〉 is the coherent state and is an eigenstate of the annihilation operator *a* for each complex number *α*. Let Φ^*Q*^ be the thermal radiation noise channel, 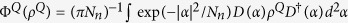, where 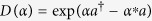 is the displacement operator, and *N*_*n*_ is the average photon number of the output state if the input is the vacuum. If the input state *ρ*^*Q*^ is a thermal noise signal with its average photon number *N*_*s*_, then the output state *ρ*^*Q*′^ will be a thermal noise signal with its average photon number *N*_*s*_ + *N*_*n*_^19^. We know that the entropy of any Gaussian state *ρ* is finite and is formulated by *S*(*ρ*) = *g*(*N*), where *g*(*x*) = (*x* + 1) ln(*x* + 1) − *x* ln *x* is a monotonically increasing convex function and *N* is the average number of photons of the Gaussian state *ρ*. Thus, we can get 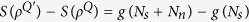. Now, we introduce a reference system *R*, initially, the joint system *RQ* is prepared in a pure entangled states 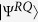 with 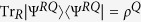, i.e., the pure state 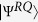 is a purification of the state *ρ*^*Q*^. The system *R* is dynamically isolated and has a zero internal Hamiltonian, while the system *Q* undergoes an internal with above thermal noise channel Φ^*Q*^. The final state of *RQ* is described by the state *ρ*^*RQ*′^. Then the entropy exchange *S*_*e*_ = *S*(*ρ*^*RQ*′^) = *g*(*N*_1_) + *g*(*N*_2_), where 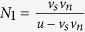, 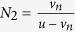, 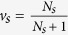, 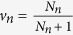
15 and *u* is the positive root of the equation 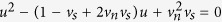.

1. If *N*_*s*_ = 0, i.e., the input state *ρ*^*Q*^ = |0〉 〈0|, then we can easily derive 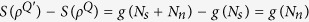. On the other hand, as *v*_*s*_ = 0 and *u* = 1, we see that *N*_1_ = 0, 
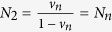
 and *S*_*e*_ = *g*(*N*_1_) + *g*(*N*_2_) = *g*(*N*_*n*_). Thus it follows that 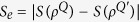 in this case.
2. If 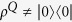, we can set *N*_*s*_ = 1 and *N*_*n*_ = 1. Then, 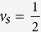 and 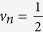. In this case we can derive 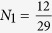 and 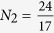. Then it is easily checked that 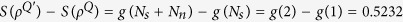 and 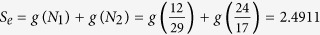. Hence we have 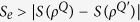 whenever *ρ*^*Q*^.

## Discussion

The notion of entropy exchange can be introduced in infinite-dimensional quantum systems with the same form as that in finite-dimensional systems if we allow it may take infinity value. Thus, for a state *ρ*^*Q*^ and a channel Φ^*Q*^ in an infinite-dimensional system *Q*, the entropy exchange *S*_*e*_ is defined as *S*_*e*_ = *S*(*ρ*^*RQ*′^), where 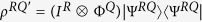 and 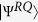 is a purification of *ρ*^*Q*^ in a larger system *RQ*. This quantity does not depend on the choice of purifications of the state *ρ*^*Q*^ and characterizes the information exchange between the system *Q* and the external world during the evolution given by Φ^*Q*^. An explicit expression for *S*_*e*_ in terms of *ρ*^*Q*^ and Φ^*Q*^ is established, which asserts that 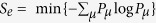, where 
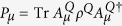
 with 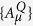 the sequence of Kraus operators in an operator-sum representation of Φ^*Q*^, and the minimum is taken over all operator-sum representations of Φ^*Q*^. In general, the entropy exchange is not equal to the change in entropy 
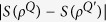
 of the system *Q*, where *ρ*^*Q*′^ = Φ^*Q*^(*ρ*^*Q*^). But we have 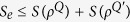, 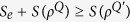 and 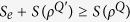. Thus, if *S*(*ρ*^*Q*^), *S*(*ρ*^*Q*′^) are both finite, then 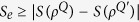. We also give some examples which illustrates that the entropy exchange is different from the change of entropy. In general the entropy exchange is larger than the change of entropy.

## Additional Information

**How to cite this article**: Duan, Z. and Hou, J. Entropy exchange for infinite-dimensional systems. *Sci. Rep.* **7**, 41692; doi: 10.1038/srep41692 (2017).

**Publisher's note:** Springer Nature remains neutral with regard to jurisdictional claims in published maps and institutional affiliations.
